# Electrospun Chitosan–Polyvinyl Alcohol Nanofiber Dressings Loaded with Bioactive Ursolic Acid Promoting Diabetic Wound Healing

**DOI:** 10.3390/nano12172933

**Published:** 2022-08-25

**Authors:** Hongyu Lv, Meng Zhao, Yiran Li, Kun Li, Shaojuan Chen, Wenwen Zhao, Shaohua Wu, Yantao Han

**Affiliations:** 1 College of Basic Medicine, Qingdao University, 308 Ningxia Road, Qingdao 266071, China; 2 College of Nursing, Qingdao University, 308 Ningxia Road, Qingdao 266071, China; 3 College of Textiles and Clothing, Qingdao University, 308 Ningxia Road, Qingdao 266071, China

**Keywords:** electrospinning, bioactive nanofibers, ursolic acid, wound dressing, diabetic wound

## Abstract

The design and development of novel dressing materials are urgently required for the treatment of chronic wounds caused by diabetic ulcers in clinics. In this study, ursolic acid (UA) extracted from Chinese herbal plants was encapsulated into electrospun nanofibers made from a blend of chitosan (CS) and polyvinyl alcohol (PVA) to generate innovative CS-PVA-UA dressings for diabetic wound treatment. The as-prepared CS-PVA-UA nanofiber mats exhibited randomly aligned fiber morphology with the mean fiber diameters in the range of 100–200 nm, possessing great morphological resemblance to the collagen fibrils which exist in the native skin extracellular matrix (ECM). In addition, the CS-PVA-UA nanofiber mats were found to possess good surface hydrophilicity and wettability, and sustained UA release behavior. The in vitro biological tests showed that the high concentration of UA could lead to slight cytotoxicity. It was also found that the CS-PVA-UA nanofiber dressings could significantly reduce the M1 phenotypic transition of macrophages that was even stimulated by lipopolysaccharide (LPS) and could effectively restore the M2 polarization of macrophages to shorten the inflammatory period. Moreover, the appropriate introduction of UA into CS-PVA nanofibers decreased the release levels of TNF-α and IL-6 inflammatory factors, and suppressed oxidative stress responses by reducing the generation of reactive oxygen species (ROS) as well. The results from mouse hepatic hemorrhage displayed that CS-PVA-UA nanofiber dressing possessed excellent hemostatic performance. The in vivo animal experiments displayed that the CS-PVA-UA nanofiber dressing could improve the closure rate, and also promote the revascularization and re-epithelization, as well as the deposition and remodeling of collagen matrix and the regeneration of hair follicles for diabetic wounds. Specifically, the mean contraction rate of diabetic wounds using CS-PVA-UA nanofiber dressing could reach 99.8% after 18 days of treatment. In summary, our present study offers a promising nanofibrous dressing candidate with multiple biological functions, including anti-inflammation, antioxidation, pro-angiogenesis, and hemostasis functions, for the treatment of hard-to-heal diabetic wounds.

## 1. Introduction

Diabetes is one of the most prevalent metabolic diseases characterized by hyperglycemia all over the world [1,2]. As a second major complication of diabetes, diabetic wounds cause significant economic burden to the society and individual patients [3,4]. As reported, the incidence rate of diabetic foot ulcer (DFU) is more than 750,000 cases annually in USA alone, and roughly 10% of them need to conduct an amputation treatment [5]. Comparing with the acute wounds caused by cutting and trauma, the chronic diabetic wound exhibits a more complicated pathological microenvironment with hard-to-heal characteristics [6]. Unfortunately, there are still no ideal strategies for the scarless regeneration and full recovery of diabetic wound in clinics [7]. The design and development of advanced dressing materials with multiple functions seem to be a promising strategy to address this intractable issue [8].

Most recently, the electrospinning technique has been widely explored to fabricate wound dressing. The electrospinning-based dressing materials have been found to exhibit some advantages compared with those traditional dressing materials such as cotton gauzes, bandages, tulles, and others [9]. The traditional fibrous dressings are commonly fabricated from fibers with diameters larger than 10 μm, which struggle to imitate collagen fibrils (50–1000 nm) existed in the native skin extracellular matrix [10,11,12]. As the control, the diameters of fibers generated from electrospinning are located in the same rang with the native collagen fibrils in skin tissues [13]. Many existing studies have demonstrated that the electrospinning nanofibers could promote cell attachment, the proliferation of human dermal fibroblasts in vitro, and the acceleration of wound healing and skin regeneration in vivo [14]. Importantly, electrospinning-based nanofibers are always collected into nonwoven mat-like structure, which has been demonstrated to possess great air and moisture permeability, as well as excellent pathogen barrier properties [15]. Therefore, electrospun nanofibrous mats are assuredly ideal candidates as wound dressing materials. Although numerous natural or synthesized polymers have been employed for the fabrication of electrospinning-based dressings, polysaccharides, such as chitosan (CS) and chitin, exhibit some outstanding characteristics, including great biocompatibility and biodegradation, as well as excellent antibacterial and hemostatic performance [16,17,18]. Unfortunately, the electrospinnability of CS is inferior, so blending with some other polymers such as polyvinyl alcohol (PVA) is commonly adopted to facilitate the electrospinning process and improve the properties of as-derived nanofiber mats [19].

Although the electrospinning-based dressings have been found to provide excellent physical cues for promoting the wound healing process, the therapeutic effect and functional restoration still need to be improved, especially for diabetic wounds [20,21]. Pathologically, the healing process of acute wounds is divided into four stages: hemostasis, inflammation, proliferation, and remodeling [21]. However, the diabetic wounds hold the healing process in the second stage and are marked by a persistent state of low inflammation, finally leading to the generation of hard-to-heal wounds [20,22]. Theoretically speaking, macrophages are critically involved in the inflammation process of wound healing [20]. Noticeably, the macrophages are induced to be transformed into the M2 (pro-healing) phenotype for accelerating angiogenesis, granulation tissue formation, and wound closure in the normal healing process [23]. However, the diabetic wounds compel the macrophage transition into the M1 (pro-inflammatory) phenotype and maintain in the M1 status for a long time, aggravating the duration and severity of the early inflammation reaction [24]. Furthermore, an imbalance of free radicals and antioxidants exists extensively in the injured tissue which ulteriorly delays the healing of diabetic wounds [25]. Therefore, decreasing ROS levels and suppressing the secretion of inflammatory cytokines are also critical elements for diabetic wound treatment. Recently, some studies demonstrated that introducing bioactive compounds into the electrospun nanofibers that can enhance the anti-inflammatory and anti-oxidant functions of electrospinning-based dressings seems to be a potentially effective route for diabetic wound treatment [26,27]. Philip et al. demonstrated that the electrospinning dressing loaded with growth factor TGF-β helped to recruit inflammatory cells to the wound site, deactivated the superoxide ions, and promoted granulation tissue formation [28]. Wu et al. found that the use of antioxidant ingredient manganese dioxide in the fabrication of dressing materials could effectively reduce the oxidative stress by eliminating ROS and replenishing oxygen to promote diabetic wound healing [29]. 

**Figure 1:**
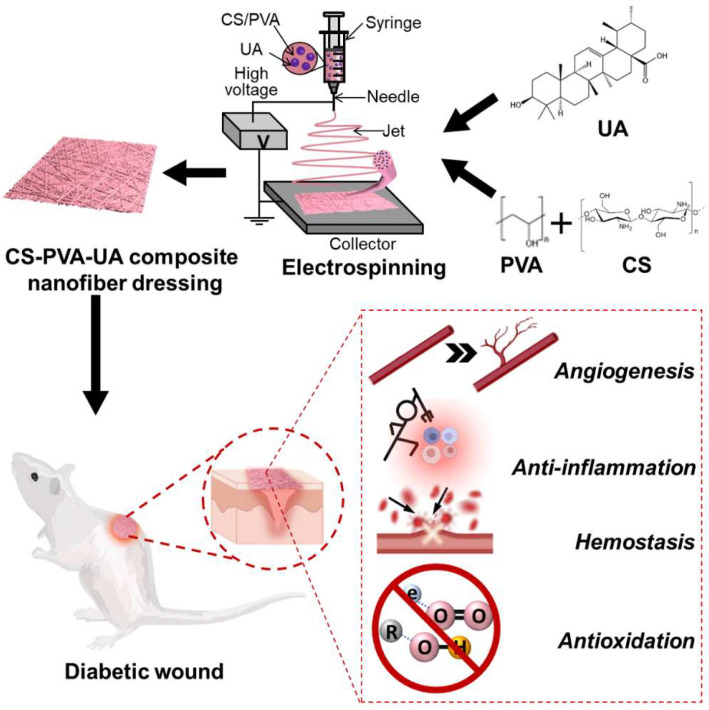
Schematic representation of the fabrication process of CS-PVS-UA nanofiber mats and their application as novel wound dressings with multiple functions including hemostasis, anti-inflammation, antioxidation, and pro-angiogenesis for the treatment of diabetic wounds.

Ursolic acid (UA) is a bioactive extract widespread in fruits and plants, possessing multiple pharmacological activities such as the ability to lower blood glucose [30,31], as well as anti-oxidation [32,33] and anti-inflammation [34,35] characteristics. UA demonstrates the protective effect on liver injury in diabetic mice by regulating the lipid metabolism, reducing oxidative stress, and enhancing the ability of anti-oxidation in liver [36]. UA also ameliorates ulcerative colitis by regulating intestinal microbiota and inflammatory cell infiltration [37]. Compared with those expensive growth factors and refractory oxide, UA showed some unique advantages including the low price, low toxicity, as well as muti-target functions [38]. In this study, UA was utilized to fabricate bioactive ingredient-contained CS-PVA nanofibrous dressings by electrospinning. The morphology, physical properties, drug release behavior, and biocompatibility of as-prepared CS-PVA-UA nanofiber mats were systematically determined. A mouse liver bleeding model was utilized to characterize the hemostatic performance of CS-PVA-UA nanofibrous mats, and the anti-inflammatory and anti-oxidative characteristics of CS-PVA-UA nanofiber mats were also verified by in vitro cell culture. Importantly, the diabetic wound model was developed in mice and was employed to investigate the skin regeneration outcomes by using our CS-PVA-UA nanofiber mats. Figure 1 shows the schematic illustration of our whole study.

## 2. Materials and Experimental Methods

### 2.1. Materials

Chitosan (CS) (M_w_ = 160 kDa, 75–85% deacetylation) was obtained from Rensin Chemicals Ltd. (Xiamen, China). Polyvinyl alcohol (PVA) (M_w_ = 146 kDa) was purchased from Qingdao Jielong Chemical Co., Ltd. (Qingdao, China). Ursolic acid (UA) was supplied by Chengdu Desite Biological Technology Co., Ltd. (Chendu, China). Acetic acid was purchased from Aladdin Reagent (Shanghai, China). All the chemical reagents were used as received without further purification.

### 2.2. Fabrication of CS-PVA and CS-PVA-UA Nanofiber Mats

Firstly, 10 mg of CS and 10 mg of PVA were dissolved into a blend solvent system made from 0.5 mL of acetic acid and 1.5 mL of deionized water to generate a homogeneous polymeric solution using a magnetic stirring apparatus. Several different concentrations of UA (0.1%, 0.2%, 0.5% (*w*/*v*)) were added into CS-PVA solution to obtain a series of UA-loaded spinning solutions, i.e., CS-PVA-0.1UA, CS-PVA-0.2UA, and CS-PVA-0.5UA. The CS-PVA solution and three different CS-PVA-UA solutions were electrospun into nanofibrous mats using a typical electrospinning device. A 21 G blunt-tip needle was utilized as a spinneret. The spinning voltage, the collecting distance between the needle tip and the metal plate, and the solution feeding rate were maintained at 15 kV, 15 cm, and 0.5 mL/h, respectively. The atmosphere temperature and relative humidity were adjusted to be 25 °C and 60% during the electrospinning process. The whole process time of electrospinning was 30 h. The as-prepared nanofiber mats were named as CS-PVA, CS-PVA-0.1UA, CS-PVA-0.2UA, and CS-PVA-0.5UA nanofiber mats according to the component and concentration differences of spinning solutions. All the nanofiber mats were vacuum-dried over night to dispose of the residual solvent.

### 2.3. Morphological Characterization

All the CS-PVA and CS-PVA-US nanofiber mats were observed with a scanning electron microscope (SEM, TESCAN VEGA3, Brno, Czech Republic). All the samples were processed with gold spraying for 90 s before observations were made. Based on the as-obtained SEM images, the average fiber diameter and fiber diameter distribution were calculated and analyzed by using an Image J software 1.8.0 (NIH, Bethesda, MD, USA).

### 2.4. Surface Hydrophilicity Measurement

The surface hydrophilicity of all the CS-PVA and CS-PVA-US nanofiber mats was determined using a water contact angle testing machine (XG-CAMD3, Shanghai, China). A 2 μL of water droplet was dropped on the surface of testing samples, and the dynamic contact angle was recorded with the time increasing.

### 2.5. Drug Release Determination

To determine the release behavior of UA from the UA-loaded CS-PVA nanofiber mats, the samples were placed in 1 mL of PBS solution and incubated at 37 °C on an incubator shaker. At pre-set time intervals, a portion of the supernatant was taken at 1, 2, 3, 4, 5, 6, 7, 8, 9, and 10 h, and measured using a UV spectrophotometer at the wavelength of 204 nm. The fresh PBS solution was then replenished to the original volume after each harvesting.

### 2.6. Biocompatibility Test

Commercial L929 cells were obtained from Chinese Academy of Sciences, Shanghai, China, and were employed to determine the biocompatibility of all the CS-PVA and CS-PVA-US nanofiber mats. The samples were immersed into commercial cell culture medium for 24 h, and the extract-contained media were harvested. L929 cells were seeded in a 96-well plate with a density of 1.0 × 10^4^ cells per well. After 24 h of culture, 100 μL of the as-harvested extract-contained medium was utilized to further culture cells for another 24 h. Then, a CCK-8 assay (Yeasen Biotechnology Co., Ltd., Shanghai, China) was conducted to quantitatively test the cell viability according to the manufacturer’s protocol. Moreover, a live–dead assay was performed to observe the cell viability from the qualitative perspective. Specifically, calcein-AM dye (Yeasen Biotechnology Co., Ltd., Shanghai, China) and propidium iodide dye (Yeasen Biotechnology Co., Ltd., Shanghai, China) were utilized to stain the live and dead cells, respectively. A confocal microscope (CLSM, Zeiss 900 CLSM, Oberkochen, Germany) was utilized to take photos of live–dead staining. The excitation wavelength and emission wavelength of Calcein AM were 490 nm and 515 nm, respectively. The excitation wavelength and emission wavelength of propidium iodide were 490 nm and 630 nm, respectively.

### 2.7. Anti-Inflammation Test

RAW264.7 cells (Chinese Academy of Sciences, Shanghai, China) were cultured to characterize the anti-inflammatory performance of all the nanofiber mats. Detailly, 1 μg/mL of lipopolysaccharide (LPS) was utilized to stimulate the as-cultured RAW264.7 cells for 24 h, and the extract obtained from the CS-PVA or CS-PVA-UA nanofiber mat was employed to culture with RAW264.7 cells. The normal cells without any treatments were employed as the control group. After 3 days of culture, the cell supernatant was harvested and two corresponding inflammatory cytokines, i.e., tumor necrosis factor-α (TNF-α) and interleukin-6 (IL-6), were detected by two ELISA kits (Sizhengbai Biotechnology, Beijing, China). In the meanwhile, the cells were stained with FITC-conjugated CD80 antibody (detecting M1 subtype, Boaoshen Biotechnology, Nanjing, China) and Cy5-conjugated CD206 antibody (detecting M2 subtype, Boaoshen Biotechnology, Nanjing, China), respectively. A FACScanTM flow cytometer (BD Biosciences, San Jose, CA, USA) was utilized to perform the flow cytometry experiment and the results were analyzed with FlowJo software 10.8.1 (Tree Star, San Carlos, CA, USA).

### 2.8. Anti-Oxidative Characterization

The dichlorodihydrofluorescein diacetate (DCFH2-DA)-labeled flow cytometry test was conducted to determine the anti-oxidative capacity of different nanofiber mats. RAW264.7 cells with a density of 1.0 × 10^6^/well were seeded and cultured in a 6-well plate overnight. Cells were pretreated with1 µg/mL of LPS for 4 h and cultured with the extracts from different nanofiber mats or *N*-acetyl-L-cysteine (NAC, ROS scavenger) for another 1 h. The normal cells without any treatments were employed as the control group. Then, the cells were incubated with 10 µM of DCFH2-DA for 30 min at 37 °C. The fluorescence intensity was recorded with a FACScanTM flow cytometer (BD Biosciences, San Jose, CA, USA), which could reflect the generation of reactive oxygen species.

### 2.9. Hemostasis Assay

A mouse model of hepatic hemorrhage was applied to determine the hemostatic capability of the as-prepared CS-PVA and CS-PVA-0.2UA nanofiber dressings. The animal studies were approved by the Qingdao University Animal Research Committee. The mouse liver was harvested and placed on a white filter paper. The liver was then punctured with an 18 G needle and the damaged liver started to bleed. The dressing sample was employed to cover the trauma site immediately [39]. The bleeding site of damaged liver was observed and recorded at 5 s, 10 s, 15 s, 30 s, and 60 s, respectively. In addition, quantitative analysis was performed by weighing the bleeding amount. The needle-punctured liver without any treatment was utilized as the control group.

### 2.10. Foundation of a Full-Thickness Wound in a Diabetic Animal Model

The in vivo animal study was approved by the Qingdao University Animal Research Committee. The mouse model with type I diabetic disease was established according to our previous studies [19,29]. The surgical procedure was performed under general anesthesia. Subsequently, 4% chloral hydrate was utilized to anesthetize the diabetic mice. The middle part of the mouse back was completely depilated, and a standardized full-thickness wound with a diameter of 10 mm was generated with a circular skin sampler. After that, a black circular rubber was sutured around the wound site to avoid the shrinkage of the mouse skin. The injured mice were divided into three groups: the control group (covered with a cotton gauze), the CS-PVA dressing group, and the CS-PVA-0.2UA dressing group. After the predetermined time intervals, the wound sites in each group were observed and recorded, and the as-obtained photographs were quantitatively analyzed using Image J software (NIH, Bethesda, MD, USA).

### 2.11. Histopathological Analysis

The tissues on the skin wound bed were collected on the 3rd, 9th, and 18th days and immersed in 4% paraformaldehyde solution (Beyotime, Shanghai, China) for 24 h. Then, dehydrated skin tissues were embedded in the paraffin and subsequently cut into slices with a thickness of 8 μm using a microtome. Hematoxylin–eosin (H&E) staining and Masson’s trichrome staining were conducted to investigate the regeneration status of the wound beds in each animal group.

### 2.12. Immunofluorescent Staining

The immunofluorescent staining was performed to observe the M1 and M2 type expression of macrophages on the wound bed by labeling CD80 (Boaoshen Biotechnology, Nanjing, China) and CD206 (Boaoshen Biotechnology, Nanjing, China) on day 3, in order to assess the in vivo anti-inflammatory function of the as-prepared different dressings. Moreover, the vascular regeneration on the wound bed was observed by staining (CD31 Boaoshen Biotechnology, Nanjing, China) on the 18th day. Primary antibodies were added dropwise and incubated overnight at 4 °C overnight, and corresponding fluorescent secondary antibodies were incubated for 30 min at room temperature. The stained samples were observed under a fluorescent microscope (Nikon, A1 MP, Tokyo, Japan), and the as-obtained images were quantitatively analyzed using Image J software 1.8.0 (NIH, Bethesda, MD, USA).

### 2.13. Statistical Analysis

Statistical analysis was carried out with ANOVA followed by post-hoc Tukey comparison. *p* < 0.05 was recognized to possess the significant difference. At least three replicates were performed for each sample in all the tests.

## 3. Results

### 3.1. Morphological Observation and Analysis of CS-PVA-UA Nanofiber Mats

**Figure 2:**
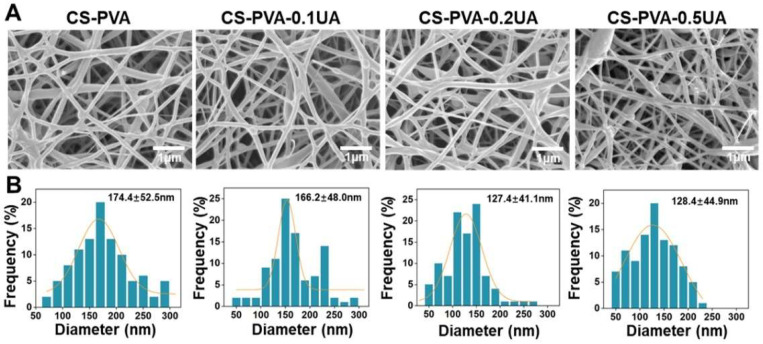
(**A**) The representative SEM images and (**B**) the fiber diameter distribution analysis of as-obtained CS-PVA, CS-PVA-0.1UA, CS-PVA-0.2UA, and CS-PVA-0.5UA nanofiber mats using a blend electrospinning strategy.

The design and development of dressing materials that can largely replicate the native skin ECM are of significant importance in skin tissue engineering, especially for the treatment of those chronic hard-to-heal wounds including diabetic ulcers [9,40]. In the present study, a blend electrospinning strategy was employed to fabricate the CS-PVA nanofiber mats, and a bioactive ingredient, UA, with different dosages was also encapsulated into CS-PVA nanofibers during the electrospinning process to generate CS-PVA-0.1UA, CS-PVA-0.2UA, and CS-PVA-0.5UA nanofiber mats, respectively. It was found that a polymeric mixture of CS and PVA possessed excellent electrospinnability, and the addition of UA did not significantly affect the spinning process. The representative SEM images of the as-prepared CS-PVA nanofiber mat and three different CS-PVA-UA nanofiber mats are presented in Figure 2A. The images show that all the nanofiber mats exhibited a nonwoven-like structure constructed with randomly oriented fibers. The results from statistical analysis show that the fiber diameter uniformity significantly decreased in the CS-PVA-0.5UA nanofiber mat (Figure 2B). In addition, the mean diameter of the CS-PVA-0.2UA and CS-PVA-0.5UA nanofiber mats was relatively lower than that of CS-PVA and CS-PVA-0.1UA nanofiber mats, indicating that the addition of UA in high doses could decrease the fiber diameter to some extent. Importantly, the mean fiber diameter of all the four different mats was in the range of 100–200 nm, which had excellent resemblance with the collagen fibrils (50–500 nm) of native skin ECM (9, 19). 

### 3.2. Biocompatibility of CS-PVA-UA Nanofiber Mats

**Figure 3:**
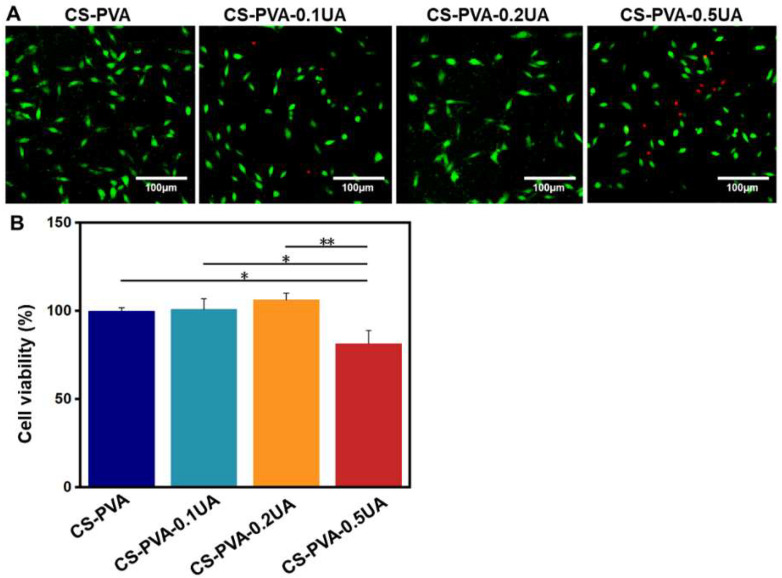
(**A**) The representative images from live–dead staining of L929 cells treated with CS-PVA, CS-PVA-0.1UA, CS-PVA-0.2UA, and CS-PVA-0.5UA nanofiber mats, respectively. Green, live cells; red, dead cells. (**B**) The quantitative viability analysis of L929 cells treated with four different nanofiber samples (*n* = 3; * *p* < 0.05, ** *p* < 0.01).

The biocompatibility of as-fabricated wound dressings is usually the first to be considered [7,9]. A commercial L929 fibroblast cell line was employed to characterize the cytotoxicity of CS-PVA, CS-PVA-0.1UA, CS-PVA-0.2UA, and CS-PVA-0.5UA nanofiber mats. The live–dead staining images show that some more dead cells stained with red color were observed in the CS-PVA-0.5UA group compared with the other three different groups (Figure 3A). The statistical results from the CCK-8 assay also confirmed that the viability of L929 cells was significantly decreased in the CS-PVA-0.5UA group (Figure 3B). In comparison, the CS-PVA, CS-PVA-0.1UA, and CS-PVA-0.2UA groups possessed high cell viability, and no significant difference was found among these three different groups. Taken together, the addition of high concentration of UA could have negative effects on cell viability to a certain degree. Therefore, the CS-PVA-0.2UA nanofiber mat was utilized as the drug-contained group for the following studies for the sake of security.

### 3.3. Surface Hydrophilicity and Drug Release Behavior of CS-PVA-UA Nanofiber Mats

**Figure 4:**
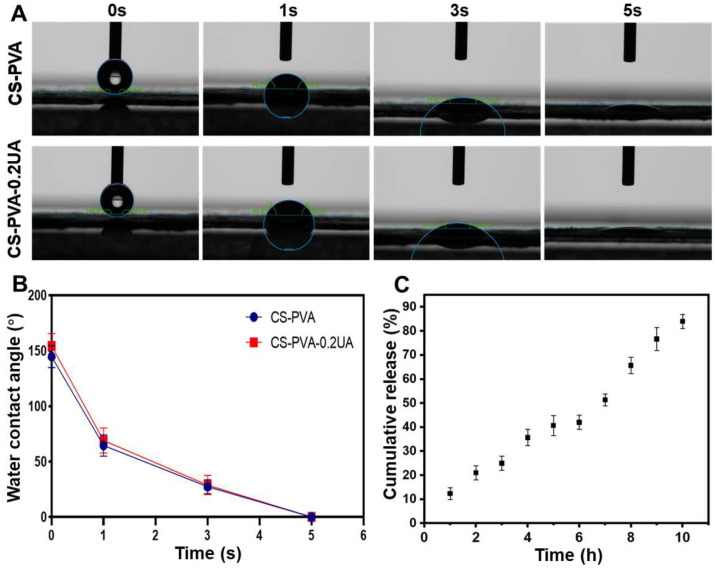
(**A**) The actual photographs and (**B**) the corresponding quantitative analysis of dynamic water contact angle of CS-PVA and CS-PVA-0.2UA nanofiber mats (*n* = 3). (**C**) In vitro accumulated release rates of UA in the CS-PVA-0.2UA nanofiber mat (*n* = 3).

The surface hydrophilicity of CS-PVA and CS-PUA-0.2UA nanofiber mats was characterized by testing and recording the water contact angle in a dynamic manner. As shown in Figure 4A,B, both the CS-PVA nanofiber mat and the CS-PUA-0.2UA nanofiber mat were demonstrated to exhibit excellent hydrophilicity and wettability with a mean water contact angle of 0°. Specifically, the water droplet could be totally absorbed in 5 s when it was applied to the surface of two different nanofiber mats. The in vitro drug release behavior of UA in the CS-PUA-0.2UA nanofiber mat was characterized by the calculation of accumulated release rates over 10 h in the PBS solution. As shown in Figure 4C, a relatively flat release curve was observed during the 10 h of incubation, and no obvious burst release phenomenon was detected. In addition, the mean accumulated release rate of UA reached 84.0% after 10 h. The stable and sustained release behavior of UA from the CS-PUA-0.2UA nanofiber mat was expected to effectively prolong the time of administration and finally improve the treatment effectiveness as a wound dressing.

### 3.4. The CS-PVA-0.2UA Nanofiber Mat Exhibited Great Anti-Inflammation and Anti-Oxidative Performances In Vitro

**Figure 5:**
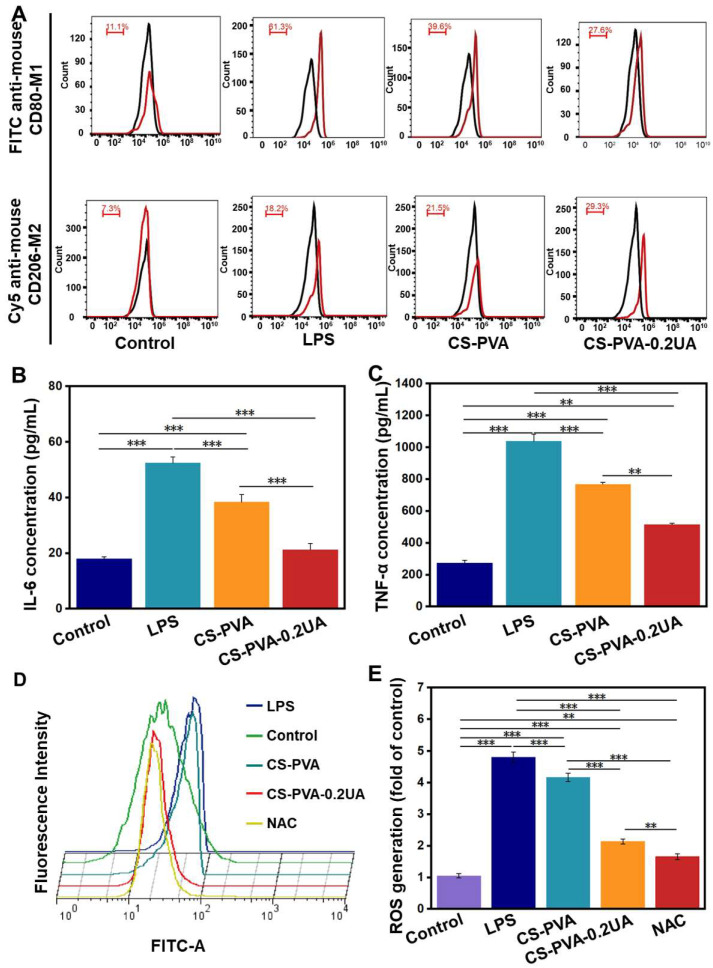
(**A**) The flow cytometry analysis of M1 subtype (CD80+) and M2 subtype (CD206+) of RAW 264.7 macrophages in different groups. The black lines and red lines stand for the normal cell reference groups and experimental cell groups, respectively. The ELISA concentration detection of (**B**) IL-6 and (**C**) TNF-ɑ secreted by RAW 264.7 macrophages in different groups (*n* = 3; ** *p* < 0.01, *** *p* < 0.001). (**D**) The qualitative analysis and (**E**) the corresponding quantitative analysis of ROS generation characterized by flow cytometry (*n* = 3; ** *p* < 0.01, *** *p* < 0.001). Control: The normal cells with any treatments; LPS: the cells activated by LPS induction; CS-PVA: LPS-activated cells treated with the CS-PVA nanofiber mat; CS-PVA-0.2UA: LPS-activated cells treated with the CS-PVA-0.2UA nanofiber mat; NAC, CS-PVA-0.2UA treated with NAC.

An ideal wound dressing should have effective inflammatory regulation function, especially for the treatment of chronic wounds caused by diabetic ulcers, given that long-term chronic inflammation is one of the key reasons to hold back the healing process of diabetic wounds [29,41]. LPS-activated RAW 264.7 cells were utilized to determine the anti-inflammatory property of the CS-PVA-0.2UA nanofiber mat. The M1 subtype of macrophages was involved in the proinflammatory process, while the M2 subtype of macrophages inhibited the inflammatory reaction [42,43]. We assumed that the RAW 264.7 macrophages induced by LPS possessed a high percentage of M2 cells. The results from the flow cytometry show that the percentage of the M1 phenotype in the LPS group was up to 61.3%. As a control, the percentage of the M1 phenotype was 39.6% in the CS-PVA group, and 27.6% in the CS-PVA-0.2UA group (Figure 5A). It was also found that the M2 phenotype percentage in the LSP group, the CS-PVA group, and the CS-PVA-0.2UA group was 18.2%, 21.5%, and 29.3%, respectively (Figure 5A). We further detected the secretion levels of two typical proinflammatory cytokines, i.e., IL-6 and TNF-α, of RAW 264.7 macrophages in four different groups. As shown in Figure 5B,C, the LPS group secreted the highest levels of IL-6 and TNF-α. In comparison, the significantly decreased expression level of IL-6 and TNF-α was found in the CS-PVA-0.2UA group. The results from both of the flow cytometry and ELISA experiments confirmed that the CS-PVA-0.2UA nanofiber mat could effectively induce the M0-to-M1 repolarization, and obviously suppress the secretion of proinflammatory cytokines. 

Some existing studies also showed that the excessive accumulation of ROS was also a negative factor used to inhibit the healing of diabetic wounds [44,45]. The ROS scavenging capacity of the CS-PVA-0.2UA nanofiber mat was also determined. A large amount of ROS was detected in the LPS-actuated RAW 264.7 macrophage group (Figure 5D,E). In comparison, the CS-PVA group exhibited a decreased ROS amount. Importantly, the ROS amount in the CS-PVA-0.2UA group was significantly lower than that of the LPS group and the CS-PVA group, but higher than that of the ROS scavenger (i.e., NAC) group. These results indicate that the addition of the UA component could increase the ROS scavenging capacity of as-prepared nanofiber mats.

### 3.5. CS-PVA-0.2UA Nanofiber Mat Possessed Excellent Hemostatic Performance

**Figure 6:**
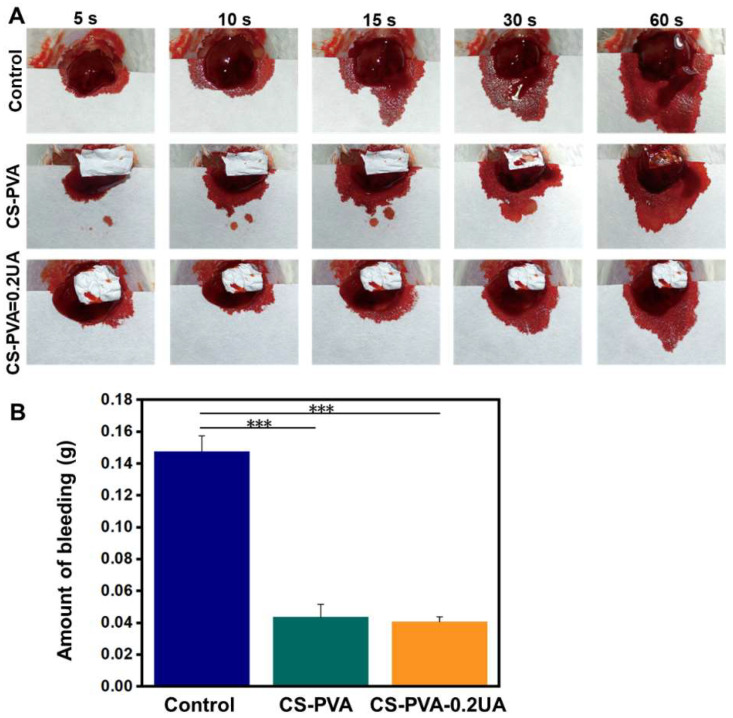
(**A**) The actual photographs of mouse hepatic hemorrhage using the CS-PVA nanofiber mat and the CS-PVA-0.2UA nanofiber mat as hemostatic materials over 60 s. Control stands for the mouse hepatic hemorrhage without any treatments. (**B**) The corresponding analysis of bleeding amount in three different groups after 60 s of bleeding (*n* = 3; *** *p* < 0.001).

The CS-PVA and CS-PVA-0.2UA nanofiber mats were further employed as hemostatic materials to investigate their hemostatic effect in the mouse hepatic hemorrhage models. As shown in Figure 6A, the bleeding diffusion area was reduced by using the CS-PVA and CS-PVA-0.2UA nanofiber mats processing the bleeding point, in comparison with the control (without any treatments). The statistical analysis shows that the mean bleeding amount after 60 s was 0.0412 g in the CS-PVA group and 0.0397 g in the CS-PVA-0.2UA group, which were both notably lower than the control group (0.146 g) (Figure 6B). Moreover, on significant difference was found between the CS-PVA group and the CS-PVA-2UA group. These results indicate that the addition of UA had no obvious effects on the hemostatic performance of nanofiber mats. The excellent hemostatic performance of nanofiber mats originated from the CS component. The previous studies have been widely demonstrated that CS could effectively promote the agglutination of red blood cells and platelet aggregation, resulting in a rapid coagulation process [19,46].

### 3.6. CS-PVA-0.2UA Nanofiber Dressing Accelerated the Closure of Diabetic Wounds

**Figure 7:**
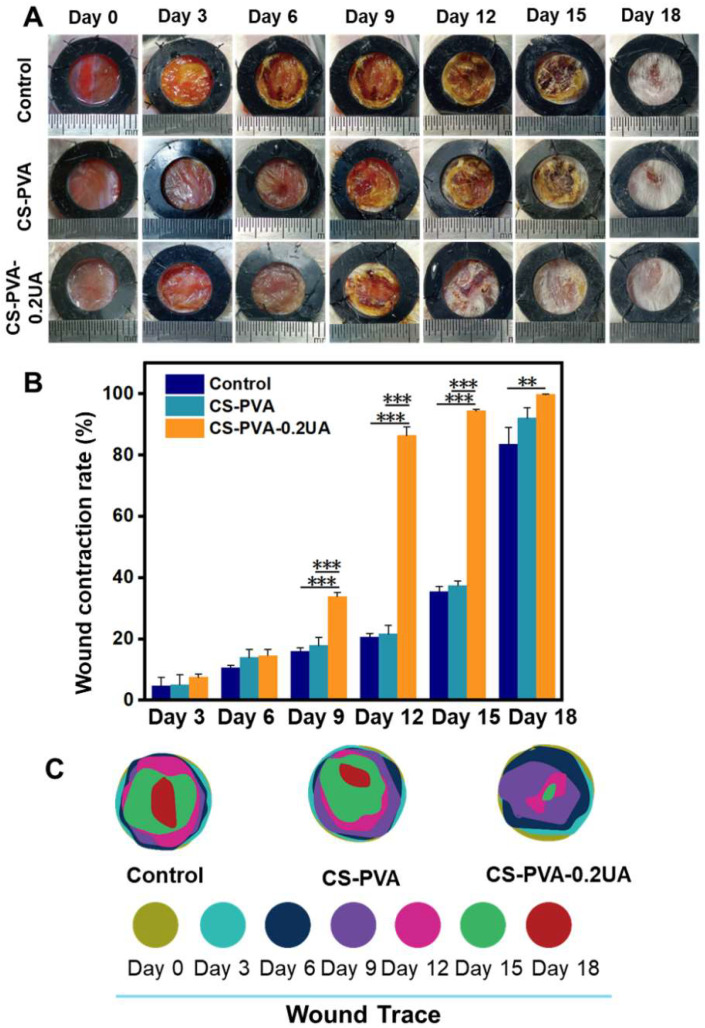
(**A**) The actual photographs and (**B**) their corresponding quantitative analysis of the dynamic wound contraction process on the wound bed in the control group, the CS-PVA group, and the CS-PVA-UA group at the predetermined time intervals (*n* = 3; ** *p* < 0.01, *** *p* < 0.001). (**C**) A simulation of the dynamic wound trace in the three different groups over the 18 days of treatment.

The full-thickness wound models were created on the backs of diabetic mice, and the CS-PVA nanofiber mat and CS-PVA-0.2UA nanofiber mat were chosen as the wound dressing candidates to investigate the treatment effects of as-prepared nanofiber mats on the diabetic wounds. Figure 7A displayed the actual photographs of dynamic changes in wound sites postoperatively in each group, and an obviously enhanced wound closure rate was found in the CS-PVA-0.2UA dressing group when compared with the control group and the CS-PVA dressing group. The quantitative analysis from Figure 7B further confirmed this conclusion. In details, the mean wound contraction rate of CS-PVA-0.2UA dressing was determined to be 86.4%, which was notably higher than the control (20.6%) and CS-PVA dressing (21.7%) on day 12 postoperatively. Moreover, after 18 days of treatment, the CS-PVA-0.2UA dressing group exhibited nearly 100% of wound contraction rate, while the wound contraction rate in the control and CS-PVA groups was only 83.6% and 92.1%, respectively. To present the wound healing process in a clearer manner, the dynamic wound trace in each group was stimulated, as shown in Figure 7C. Overall, the CS-PVA-0.2UA nanofiber dressing was demonstrated to accelerate the wound healing process in diabetic mice.

### 3.7. CS-PVA-0.2UA Nanofiber Dressing Suppressed the Inflammation and Promoted the Re-Epithelialization during the Healing Process of Diabetic Wounds

**Figure 8:**
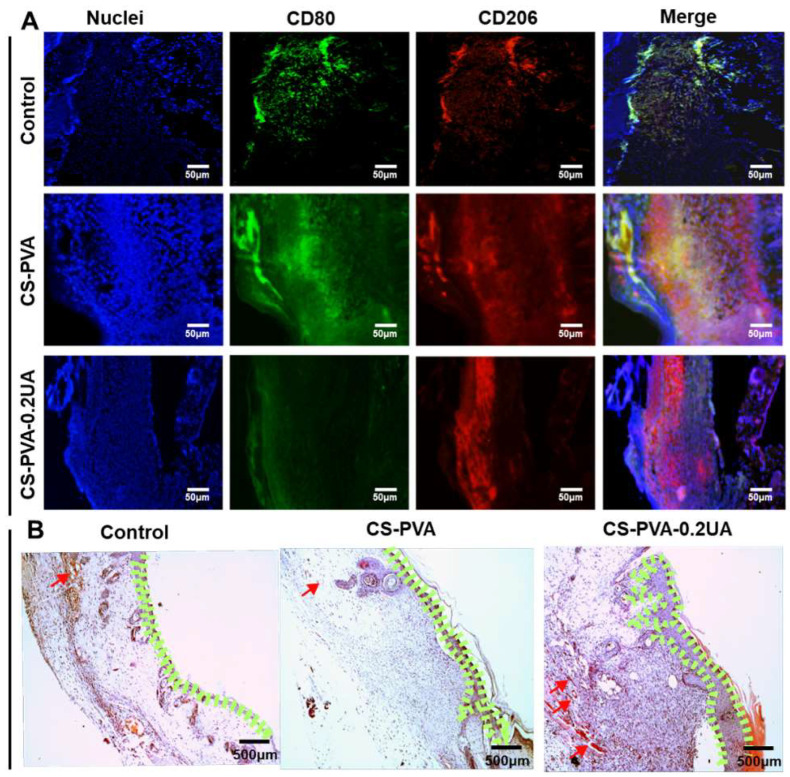
(**A**) The representative immunofluorescent staining images of M1 macrophages (marker CD80) and M2 macrophages (marker CD206) in the regenerated tissues on the wound bed for the control group, the CS-PVA group, and the CS-PVA-UA group on day 3 postoperatively. (**B**) The representative H&E staining images of as-formed granulation tissues on the wound bed for the control group, the CS-PVA group, and the CS-PVA-UA group on day 9 after surgery. The area closed with green lines strands for the re-epithelialization layer, and the red arrows stand for the newly formed blood vessels.

The macrophage is a prominent inflammatory cell and plays a crucial role in the early inflammatory stage of wound healing [29,47]. Compared with the normal wounds, the macrophages in the diabetic wounds commonly exhibited a high expression level of the pro-inflammatory M1 phenotype, rather than the pro-healing M2 phenotype, which suppressed the transition from the inflammatory phase to the proliferative phase, finally leading to the generation of chronic hard-to-heal wounds [24,48]. Moreover, some previous studies indicated the inflammatory situation was especially serious on day 3 after the wound was created [47]. In our study, the regenerated tissues on the wound bed were collected on day 3, and the distributions of macrophage subtypes were detected via immunofluorescent staining. As shown in Figure 8A, CD80 and CD206 were selected as the M1-specific marker and M2-specific marker, respectively. Large amounts of M1 macrophages were observed to be aggregated on the wound site in both the control group and the CS-PVA dressing group. As controls, the CS-PVA-UA group showed less expression of M1 macrophages, but the largely increased expression of M2 macrophages. These results indicate that the CS-PVA-UA nanofiber dressing could regulate the transition of M1 phenotype into M2 phenotype of macrophages, further promoting the transition of the pro-inflammatory phase into the pro-healing phase during the healing process of diabetic wounds. 

The proliferation and remodeling behavior of regenerated tissues on the wound bed were investigated on day 9 postoperatively. Figure 8B displays that a small quantity of neo-epidermis region was observed in the control group, while a large quantity of epidermis-like region was formed in both of the CS-PVA dressing group and the CS-PVA-0.2UA dressing group. Importantly, the thickness of re-epithelialization layer in the CS-PVA-0.2UA dressing group was larger than the CS-PVA dressing group. It should be noticed that a small amount of blood vessels was sparsely found in all the three different groups.

### 3.8. CS-PVA-0.2UA Nanofiber Dressing Promoted the Collagen Deposition, Angiogenesis, and Regeneration of Hair Follicles during the Healing Process of Diabetic Wounds

**Figure 9:**
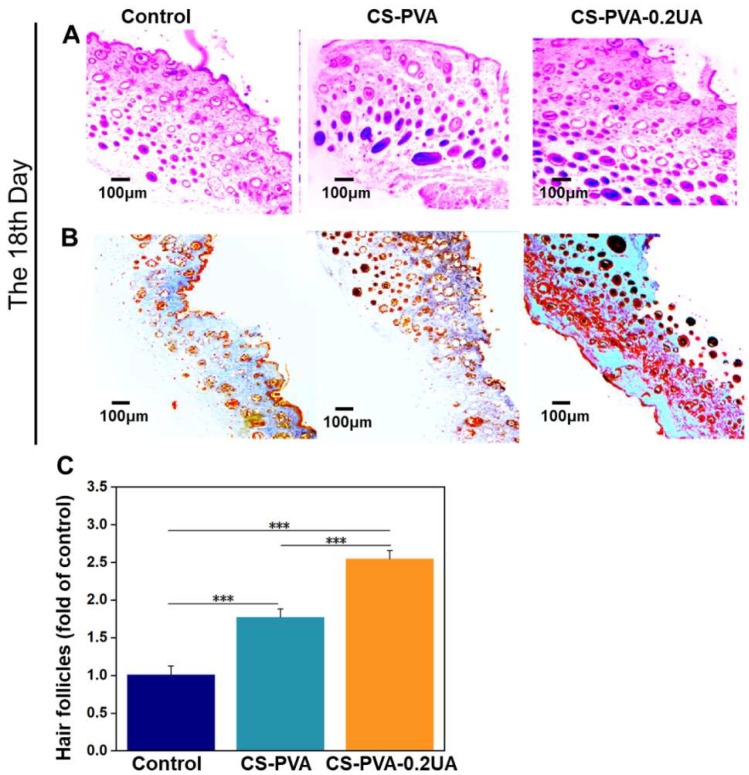
(**A**) The representative H&E staining images and (**B**) the representative Massion’s trichrome staining images of as-regenerated skin tissues on the wound bed for the control group, the CS-PVA group, and the CS-PVA-UA group on day 18 after surgery. (**C**) The corresponding analysis of as-regenerated hair follicles in three different groups after 18 days of treatment (*n* = 3; *** *p* < 0.001).

The results from Figure 7 demonstrate that the diabetic wounds treated with our CS-PVA-0.2UA nanofiber dressings exhibited a nearly complete wound healing at day 18. However, the quality of regenerated tissues on the wound bed still remained unknown. The histological analysis of as-harvested skin tissues on the wound bed on day 18 was performed in this section. As already established, the hair follicle is an essentially important cutaneous appendage, and it can be selected as an effective indicator for the characterization of functional recovery of regenerated skin tissues [9,29]. Figure 9A,C show that the CS-PVA-0.2UA dressing group possessed the highest regeneration rate of hair follicles, and the CS-PVA dressing group exhibited the second highest one, which were both far more than the control group on day 18 postoperatively. The secretion and deposition of collagen is also an important indicator for the quality detection of as-regenerated skin tissues [6,7,9]. The results from Masson’s trichrome staining displayed that the CS-PVA-0.2UA dressing group possessed the highest collagen rates in the regenerated skin tissues on the wound bed after 18 days of treatment (Figure 9B).

**Figure 10:**
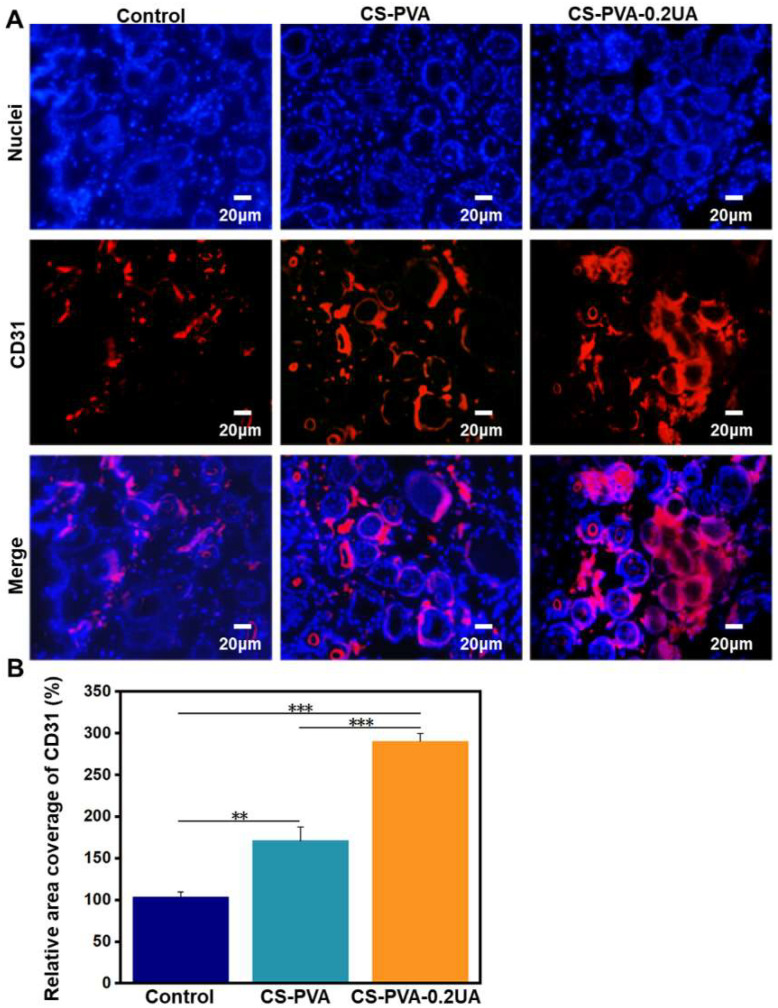
(**A**) The representative immunofluorescent staining images of CD 31 (a neovascularization marker) in the regenerated tissues on the wound bed for the control group, the CS-PVA group, and the CS-PVA-UA group on day 18 postoperatively. (**B**) The corresponding analysis on the coverage area of CD31-positive tissues in three different groups after 18 days of treatment (*n* = 3; ** *p* < 0.01, *** *p* < 0.001).

The vascularization in the regenerated skin tissues also plays essentially important roles in the wound healing, which can provide the necessary required nutrition during the wound healing process [46]. Some previous studies claimed that the limited neovascularization was a negative factor that should be addressed for the treatment of diabetic wounds [49,50]. To evaluate the vascularization of three different research groups on day 18 postoperatively, the immunofluorescent staining of CD31 (a neovascularization marker) was conducted. As shown in Figure 10A, compared with the control group and the CS-PVA dressing group, more positive staining of CD31 with strong red fluorescence was expressed in the CS-PVA-0.2UA group. Moreover, quantitative results in Figure 10B further reveal that a remarkable three-fold increased coverage area of CD31-positive tissue was observed in the CS-PVA-0.2UA group compared to the control group, indicating that the UA exhibited a high angiogenesis-promoting effect that facilitated the formation of new blood vessels in the regenerated skin tissues. In summary, our CS-PVA-0.2UA nanofiber mat-constructed dressing could promote and accelerate the high-quality healing process of diabetic wounds. As a fundamental study, some further studies on UA stability, dressing degradation, and shelf life, for example, as well as preclinical animal models, should be carried out to confirm the performances of our CS-PVA-UA nanofiber dressings before clinical use.

## 4. Conclusions

In our present study, a series of nanofiber mats were successfully fabricated by electrospinning a blend of CS and PVA, and appropriately adding UA. The as-prepared CS-PVA-UA nanofiber mats were demonstrated to possess great morphological resemblance to the collagen fibrils which exist in the native skin ECM, good surface hydrophilicity and wettability, as well excellent hemostatic performance. The moderate addition of UA into the CS-PVA nanofibers was also found to possess great biocompatibility and sustained drug release properties. The results from both of in vitro and in vivo studies indicate that the wound dressings constructed with CS-PVA-0.2UA nanofiber mats could effectively suppress inflammation and oxidative stress, while significantly promoting the angiogenesis, collagen deposition, re-epithelialization, and regeneration of hair follicles, thus resulting in the rapid and high-quality regeneration and healing process of skin wounds in diabetic mice. Overall, our present study offers a simple and feasible routine for the design and development of innovative CS-PVA-UA nanofiber dressings, demonstrating the huge potential of multifunctional dressing materials for the treatment and repair of hard-to-heal diabetic wounds.

## Data Availability

The data presented in this study are available on request from the corresponding author.
